# A Comprehensive Review of Thyroid Eye Disease Pathogenesis: From Immune Dysregulations to Novel Diagnostic and Therapeutic Approaches

**DOI:** 10.3390/ijms252111628

**Published:** 2024-10-29

**Authors:** Merve Kulbay, Stuti M. Tanya, Nicolas Tuli, Jade Dahoud, Andrea Dahoud, Fares Alsaleh, Bryan Arthurs, Christian El-Hadad

**Affiliations:** 1 Department of Ophthalmology & Visual Sciences, McGill University, Montreal, QC H4A 0A4, Canada; merve.kulbay@mail.mcgill.ca (M.K.); stuti.tanya@mail.mcgill.ca (S.M.T.); andrea.dahoud@mail.mcgill.ca (A.D.); fares.alsaleh@mail.mcgill.ca (F.A.); bryan.arthurs@mcgill.ca (B.A.); 2 Faculty of Medicine and Health Sciences, McGill University, Montreal, QC H3G 2M1, Canada; nicolas.tuli@mail.mcgill.ca; 3 Faculty of Medicine, University of Montreal, Montreal, QC H3T 1J4, Canada; jade.dahoud@umontreal.ca

**Keywords:** thyroid eye disease, grave’s orbitopathy, grave’s disease, grave’s pathophysiology, deep learning for thyroid eye disease, immunotherapy for grave’s disease

## Abstract

Thyroid eye disease is a complex inflammatory disorder of the orbit that has gained tremendous interest over the past years, and numerous scientific efforts have been deployed to elucidate its pathophysiology for novel drug development. Our manuscript will delve into the molecular dysregulations involved in the pathogenesis of thyroid eye disease that led to its clinical manifestations. Abnormalities within the apoptotic pathway, inflammatory cascade, and autoimmune regulatory systems will be covered. We will further discuss the challenges involved in its diagnosis and management and provide a summary of the current diagnostic tools (i.e., molecular biomarkers, diagnostic scores) from the perspective of clinicians. Finally, our comprehensive literature review will provide a thorough summary of most recent preclinical and clinical studies around the topic of thyroid eye disease, with an emphasis on the manuscripts published within the last five years. We believe our manuscript will bring novelty within the field by bridging the fundamental sciences with the clinical aspect of this disease. This review will be a great tool for clinicians in better understanding the pathogenesis of thyroid eye disease while providing an outlook on future perspectives (i.e., liquid biopsies, artificial intelligence).

## 1. Introduction

Thyroid eye disease (TED), also known as thyroid-associated ophthalmopathy or Grave’s orbitopathy (GO), represents the most common extrathyroidal manifestation of Grave’s disease (GD), but may also be seen in other thyroid disorders such as Hashimoto’s thyroiditis (HT) [1]. TED is believed to affect 25–50% of patients with GD and has an annual incidence of 20 cases per 100,000 people [2]. Nearly 5% of patients with TED will further develop sight-threatening complications, such as dysthyroid optic neuropathy (DON) or exposure keratopathy, therefore requiring a surgical intervention in moderate to severe cases [3]. The economic surgical burden of TED was shown to reach approximately 43.5 million dollars in the United States based on the National Ambulatory Surgery Sample (NASS) database [3]. Furthermore, TED was shown to severely impact the quality of life of affected (QOL) individuals; by using the Grave’s Ophthalmopathy QOL instrument, it was shown that individuals who exhibit ocular pain, diplopia, and blurry vision report a lower QOL [4]. The presence of bothersome symptoms in TED patients was shown to be linked to feelings of depression and anxiety and a decline in self-confidence [5]. TED nevertheless comes with a non-negligible economic and psychosocial burden.

TED is characterized by an inflammatory orbital process and remodeling of surrounding connective tissue, with subsequent fat accumulation, myositis, and/or tissue scaring due to autoimmune activation of orbital fibroblasts [6,7]. It is a complex clinical entity with a growing body of literature on its pathogenesis, emerging molecular biomarkers, and novel therapeutics [2,8]. Elucidating the pathophysiology of TED is the cornerstone of evolving diagnostic criteria, drug development, and surgical management. Therefore, this article provides an overview of the pathogenesis of TED and upcoming developments in diagnostic and therapeutic approaches. By bridging the fundamental science to the clinical aspect of TED diagnosis and management, this comprehensive literature review is a crucial tool for many healthcare professionals.

## 2. Pathogenesis

**Figure 1:**
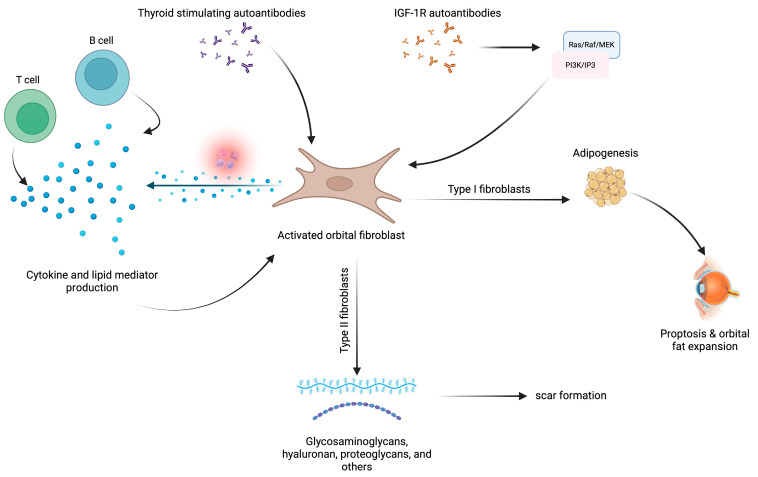
Schematic representation of thyroid eye disease pathophysiology. The backbone of TED pathogenesis involves the uncontrolled activation of orbital fibroblasts by thyroid-stimulating autoantibodies, IGF-1R autoantibodies, and through the proinflammatory microenvironment created by T- and B-cells. Activated orbital fibroblasts lead to adipogenesis, which is involved in proptosis and orbital fat expansion, as well as glycosaminoglycan, hyaluronan, and proteoglycan production, markers involved in scar formation. The figure was created with BioRender.com.

The backbone of TED pathogenesis involves the uncontrolled activation and proliferation of orbital fibroblasts, a multifactorial process (Figure 1). Activated orbital fibroblasts can differentiate into two distinct phenotypes: lipid-rich adipocytes or scar-forming myofibroblasts [6]. This differential is crucial given that clinically, they form two distinct disease entities, which are type I and type II TED, respectively. The ensuing inflammatory process is a result of extracellular matrix production by activated orbital fibroblasts, as well as chemotaxis of B- and T-cells, mast cells, and macrophages to the orbital tissue [9,10,11,12]. Throughout the body of molecular pathways that have been proposed to be involved in orbital fibroblast activation, immune dysregulations are the most important. In this section, we will review the key features involved in TED pathogenesis while discussing the most recent advances. 

### 2.1. Autoantibodies

The production of autoantibodies directed towards the thyroid stimulating hormone (TSH) receptor (TSHR) and insulin-like growth factor-1 receptor (IGF-1R) constitutes the main key factors involved in orbital fibroblast activation and proliferation [13,14]. A multicenter study amongst pediatric patients demonstrated that the level of TSHR autoantibodies in individuals with GD correlated with disease severity and extrathyroidal manifestation [15]. Conversely, immunoglobulins (Igs) were shown to stimulate IGF-1R in patients with GD; depletion of serum Igs significantly decreased IGF-1R in patients with GD [16]. However, the importance of IGF-1R in TED pathogenesis is yet controversial, with different works confirming or refuting the presence of IGF-1R autoantibodies in GO [17,18,19,20].

TSH-R is a G-protein-coupled transmembrane receptor with major functions in cell proliferation [21]. Using orbital fibroblasts from patients with TED, it was shown that in vitro TSH stimulation significantly increased cell proliferation through the direct induction of the phosphoinositide 3-kinase (PI3K) pathway, and indirect upregulation of the micro-RNAs (miRNA) miR-146a and miR-155 expression was also reported [22]. The activation of the PI3K pathway in orbital fibroblasts leads to cyclic adenosine monophosphate (cAMP) production and subsequent secretion of hyaluronan [23].

The IGF-1R is a member of the tyrosine kinase class of membrane receptors [24]. Its expression was shown to be significantly elevated in orbital fibroblasts and T- and B-cells of individuals with TED [25,26,27,28]. IGF-1R leads to downstream activation of the Ras/Raf/MEK and PI3K signaling pathways, therefore leading to cell proliferation, differentiation, inflammation, adipogenesis, and scar formations—hallmarks in TED [6]. Furthermore, once activated by its ligand, it was shown that IGF-1R localizes in the nucleus of orbital fibroblasts through ADAM17 signaling [25]. Recently, Wang et al. have demonstrated the crucial role of IGF-1R in cellular communication and interaction between orbital fibroblasts and B-cells [26]. Orbital fibroblasts highly expressive in IGF-1R were cocultured with peripheral B-cells, resulting in an observed elevation in the expression of interleukin (IL)-6 and RANTES [26]. The authors also observed significant inhibition of the inflammatory process when pretreating the cells with rituximab [26]. Conversely, the role of IGF-1R in T-cell activation is yet to be fully elucidated in TED. A recent study performed by Kiernan et al. demonstrated that IGF-1R signaling through IGF-1 binding induces Th17 T cell subtype activation, with subsequent IL-17 production and a decrease in reactive oxygen species (ROS) production within Th17 cells [29]. The role of Th17 cells in GO pathogenesis has been underlined in the literature; Th17 cells promote orbital fibroblast and CD34+ fibroblast differentiation, adipogenesis, and orbital fibrosis in patients with GO [30,31].

### 2.2. Immune Dysregulations Leading to Inflammation, Adipogenesis, Myofibrillogenesis, Hyaluronan Synthesis, and Scarring

The role of lipid mediators (e.g., prostaglandins (PGs)) and cytokines (e.g., IL-1β, IL-6, IL-15, IL-17, tumor necrosis factor (TNF)α, platelet-derived growth factor (PDGF), and transforming growth factor (TGF-β)) in TED pathogenesis have been reported on numerous occasions in various comprehensive literature reviews and studies [6,13,32,33,34,35,36]. This ensuing section reviews the most novel advances in the field while providing the fundamental concepts on TED pathogenesis. We suggest referring to the provided references for further details. 

The underlying pathogenesis of TED involves an autoimmune response in which B-cells, T-cells, and CD34+ fibrocytes infiltrate the orbits following TSH-R and IGF1-R stimulation by autoantibodies [37]. The lymphocytes facilitate orbital fibroblast proliferation and differentiation through the release of pro-inflammatory cytokines and chemokines, ultimately leading to additional orbital fibroblast differentiation, adipogenesis, myofibrillogenesis, hyaluronan synthesis, and finally tissue scarring if left untreated (Figure 1) [18].

A fundamental step in the management of TED is to better characterize the pathogenesis of TED and factors inducing the activation of orbital fibroblasts. Recently, it was demonstrated that orbital fibroblast activation was under the regulation of SOX9; SOX9 was shown to activate orbital fibroblasts through the upregulation of mitogen-activated protein kinase (MAPK)/extracellular signal-regulated kinase (ERK) 1/2 pathways [38]. Furthermore, orbital fibroblast adipogenesis was observed to be under the regulation of the Piezo1 receptor, a mechanosensitive receptor. It is hypothesized that following increased intraorbital pressure due to extraocular muscle enlargement, Piezo1 receptors, found in orbital fibroblasts, are activated and inhibit adipogenesis of fibroblasts, as characterized by decreased expression of peroxisome proliferator-activated receptor gamma (PPARγ) and CEBPα mRNAs [39]. Conversely, Yes-associated protein (YAP), another mechanosensitive receptor, was recently shown to be involved in chronic fibrosis in GO; pharmacological inhibition of YAP in primary cultured orbital fibroblasts was shown to significantly decrease TGFβ-induced myofibroblast differentiation and subsequent collagen formation [40].

## 3. Clinical Features

**Figure 2:**
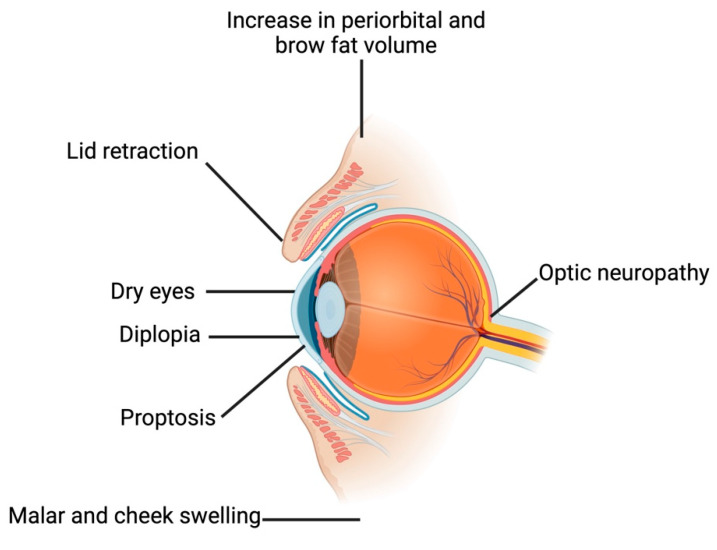
Schematic representation of the clinical manifestations of thyroid eye disease. The most common clinical symptoms involved in thyroid eye disease are depicted in this illustration. The figure was created with BioRender.com.

Lid retraction, proptosis, and diplopia are hallmarks of TED (Figure 2), although many patients initially present with more elusive and milder signs and symptoms such as chemosis, dry eyes, or soft-tissue swelling [41]. Inflammation and expansion of the fibroadipose and extraocular muscle (EOM) tissues facilitated by the TSHR-Ab/IGF-1R signaling complex underlies the eyelid retraction, proptosis, diplopia, and the dreaded but rare DON seen in TED [1,42,43]. Changes associated with thyroid disease beyond the orbit include an increase in periorbital and brow fat volume, lateral flare of the eyebrow, and malar and cheek swelling, signifying a diffuse facial soft tissue expansion [44]. TED often presents bilaterally, but unilateral or asymmetric disease occurs as well. It is reported as having a higher incidence in women yet presents with greater severity and at an older age in men [45]. Pediatric patients are rare, with typically milder disease compared to adults [1]. 

**Figure 3:**
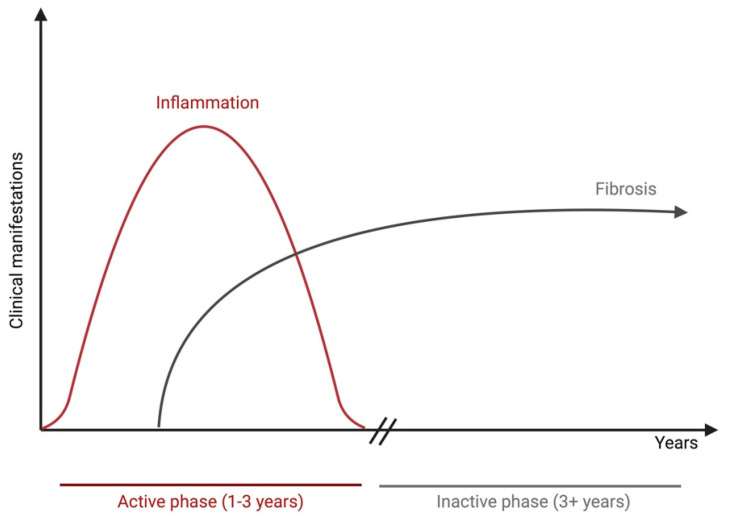
Schematic representation of the Rundle’s curve. The thyroid eye disease course is composed of an active phase, which lasts from 1 to 3 years and may or may not involve resolution of clinical manifestations during the disease course, and an inactive phase of 3 years duration and more. The figure was created with BioRender.com.

TED is classically described as active or inactive and follows the trajectory of the Rundle’s curve (Figure 3). The active phase constitutes progressive inflammation lasting between 6 and 24 months, ultimately reaching a plateau, at which point the inactive phase commences with fibrosis and regression of inflammation [42]. Reactivation of TED can occur in certain circumstances. Several studies have shown that smoking constitutes one of the major risk factors for TED reactivation [46,47,48]. Smokers with TED were also shown to be at greater risk for TED progression, as well as poor treatment outcomes [46]. Additional risk factors for disease reactivation encompass sustained TSH-R antibodies [14], radioactive iodine treatment (risk of progression of 15–39%) [49,50], and steroid treatment discontinuation in moderate-to-severe TED patients, in which up to 25% of reactivation rates were reported [51,52].

The most common finding is upper eyelid retraction, occurring in 90% of patients. The etiology of upper lid retraction is believed to be multifactorial, with sympathetic overactivity, inflammation, and fibrosis of Muller’s muscle, enlargement and overactivity of the levator palpebrae superioris (LPS) secondary to inferior rectus restriction by way of Hering’s law, and LPS overaction secondary to weakening of the orbicularis oculi muscle. The etiology of lower lid retraction is believed to be in part due to tension on the lid margin from inferior rectus enlargement [42]. Eyelid edema may be attributed to lymphatic dilatation and perivascular lymphocytic infiltrates in the dermis [42].

Depending on the severity of lid retraction, chronic eye exposure may develop, leading to dry eye disease (DES) and, if severe, exposure keratopathy with a risk of corneal scarring, ulceration, perforation, and endophthalmitis [53,54]. This is further exacerbated by the presence of lagophthalmos, decreased blink frequency, and increased tear evaporation [53,54]. In addition to an evaporative dry eye component, there are also aqueous and lipid deficiencies in TED, with decreased tear production and increased meibomian gland dropout [55]. Inflammatory changes in the ocular surface, including squamous cell metaplasia, superior limbic keratoconjunctivitis, and increased pro-inflammatory cytokines such as IL-1β, IL-6, IL-7, L-8, IL-10, IL-17A, TNF-α, lysozyme C, lacritin, and zinc-alpha-2 glycoprotein 1, with subsequently decreased tear breakup time and Schirmer’s test, have been reported [55,56,57,58,59,60,61]. Occasionally, inflammatory DES may be an early symptom of TED; in fact, one single-center study found that nearly 4% of patients with refractory DES had occult TED [62]. Treatment options for DES secondary to TED include lubrication, topical cyclosporine, topical steroids, and tarsorrhaphy [63]. 

Proptosis affects 60% of patients and is caused by the expansion of EOM and orbital fat, further increasing the risk of eye exposure [53,54] EOM dysfunction, observed in 40% of patients, manifests itself as diplopia and misalignment of the eyes [53]. The inferior rectus, then the medial rectus, and ultimately the superior rectus are most commonly involved, with only rare involvement of the lateral rectus or oblique muscles [64]. The resultant restrictive strabismus, occurring in 15% of patients, manifests as hypotropia and esotropia, which last beyond the active phase of TED due to eventual collagen deposition and fibrosis [65]. 

TED mainly causes diplopia during upgaze due to inferior rectus muscle hypertrophy, but in some patients, diplopia during downgaze caused by superior rectus hypertrophy is particularly debilitating for daily activities [59]. Diplopia from strabismus may be managed with prisms, monocular occlusion, and botulinum toxin A injection into the affected EOMs, and eventual strabismus surgery once the disease is rendered inactive [53,54,66]. Eye pain, described as dull and pressure-like, is reported in 30% of patients [53]. 

Dyschromatopsia, visual field loss, and/or visual acuity loss due to DON occurs in 6–15% of patients and is an emergency requiring immediate management, including surgical orbital decompression [53,67]. The mechanism of action for DON includes mechanical compression of the optic nerve by the EOMs, an increase in orbital adipose tissue, crowding of the orbital apex, venous congestion, and resultant ischemia. A component of stretch optic neuropathy from proptosis has also been hypothesized [68]. Older age, male sex, smoking, TSHR-Ab levels, and the presence of the cytotoxic T-lymphocyte-associated antigen-4 (CTLA-4) gene are identified as potential risk factors [69]. Additionally, Asian patients, who often present with more diffuse EOM involvement yet less prominent proptosis, may be at a greater risk of DON, possibly due to the shallower and more compact orbital anatomy [67]. Indeed, patients with minimal proptosis were found to be at higher risk of neuropathy due to the restrictive space within the bony walls, leading to a secondary ‘compartment syndrome effect’ [54]. 

A recent meta-analysis has demonstrated an association between patients with TED and the incidence of glaucoma; approximately 29% of patients with TED had glaucoma in comparison to non-TED patients [70]. Furthermore, the odds ratio (OR) for glaucoma diagnosis in patients with TED was reported to be 6.42 (95% confidence interval 4.76–8.70). However, previous results on this topic are conflicting. Kim et al. previously reported a similar incidence of glaucoma in TED patients when compared to the general population but a greater prevalence (6.8%) of ocular hypertension in TED patients [71].

The increased risk of open-angle glaucoma (OAG) can partially be due to the elevated episcleral venous pressure and changes in ocular perfusion; thus, glaucomatous optic neuropathy must be distinguished from DON [59,69]. The pathophysiology of TED-associated glaucoma remains complex. Alongside changes in episcleral venous pressure, deposition of glycosaminoglycans within the trabecular meshwork was also hypothesized to be involved in its pathophysiology [72]. In addition, the treatment of TED, which is further discussed in the ensuing sections, can raise the intraocular pressure, therefore establishing different pressure gradients within the lamina cribrosa and leading to glaucomatous changes [73]. Nevertheless, additional studies are required to better establish the relationship of TED with the prevalence or incidence of glaucoma in this population.

### 3.1. Risk Factors

#### 3.1.1. Metabolic and Environmental Risk Factors

Hyperthyroidism—and, to a much lesser extent, hypothyroidism—exacerbates TED; thus, achieving euthyroid status is essential to stabilizing the disease process. In fact, the onset of hyperthyroidism correlates with the onset of TED within 18 months before or after in 85% of cases [59]. Smoking is a significant modifiable risk factor—smokers with GD have nearly a 10-fold higher risk of developing TED compared to non-smokers, tend to develop more severe forms of TED, and respond less favorably to immunosuppressive treatments; these sequelae are diminished in former smokers. Hypoxia and oxidative stress from active smoking are believed to be the culprits [1,31,67]. Recent studies have explored the role of hypercholesterolemia in TED development, with some suggesting a beneficial effect of statins, although the mechanism of both hypercholesterolemia and statins in the role of TED is not well understood [1]. Diabetes, obstructive sleep apnea, and vitamin D deficiency have also been implicated as potential risk factors [67].

#### 3.1.2. Genetic Risk Factors 

Although genetic predispositions have been stipulated to be involved in the differences observed for the susceptibility for TED development in different ethnic populations, until today, this aspect remains poorly understood [74]. Single nucleotide polymorphisms (SNPs) in the genes coding for peroxisome proliferator-activated receptor gamma (PPARγ) and TSH-R were proposed as one of the mechanisms [75,76,77].

## 4. Diagnostic Methods

### 4.1. History and Ocular Exam

Patient history, including age, sex, ethnicity, comorbidities, systemic associations, pregnancy or pregnancy potential, tobacco smoking, other environmental and psychological stressors, and likelihood of adherence to medical therapies, must be elicited in any new patient with TED. It is important to ensure the absence of overt thyrotoxicosis, which can be life-threatening with complications including ischemia, atrial fibrillation, congestive heart failure, thromboembolism, stroke, psychosis, paralysis, and thyroid storm [43].

A comprehensive ocular examination includes documentation of visual acuity, presence or absence of a relative afferent pupillary defect, intraocular pressure, and color vision (the Hardy–Rand–Rittler plates may be more sensitive than the Ishihara plates for DON, given it would result in an acquired color vision deficiency) [78], Hertel exophthalmometry to document proptosis, and extraocular movements to identify limitations (Table 1). Eyelid changes, including the presence of lateral flare, lid lag, lagophthalmos, Bell’s reflex, and upper and lower eyelid retraction, should be noted. Ocular surface changes, including tear breakup time or Schirmer’s test, presence of superior limbic keratoconjunctivitis, exposure keratopathy, and corneal ulceration, and any related sequelae such as corneal perforation must be noted [59]. Many providers will obtain optical coherence tomography (OCT) of the macula and optic nerve as well as a formal visual field to document baseline structure and function; this is especially pertinent in cases where glaucomatous optic neuropathy—and, of course, DON—may be suspected.

### 4.2. Assessment of Clinical Activity

Several classification systems to quantify the clinical activity and severity exist and are summarized in Table 2 [59,79]. The NOSPECS and RELIEF classification systems represent now obsolete classification systems historically used to quantify clinical severity without information on clinical activity [43,59,67,79]. The CAS (Clinical Activity Score) grading system assesses clinical activity with a 7-point system for initial evaluation. Successive examinations are scored with a 10-point system. A score greater than 3/7 or 4/10 is considered an active disease. The EUGOGO (European Group of Graves’ Orbitopathy) severity grading system assesses clinical activity and severity with mild, moderate-to-severe, and sight-threatening categories. Mild disease comprises one or more of the following: minor lid retraction < 2 mm, mild soft-tissue involvement, exophthalmos < 3 mm above normal for sex and race, and transient or no diplopia. Moderate-to-severe disease comprises one or more of the following: lid retraction > 2 mm, moderate-to-severe soft-tissue involvement, exophthalmos > 3 mm above normal for sex and race, and inconstant or constant diplopia. Finally, sight-threatening disease includes DON, or corneal breakdown. The VISA (Vision, Inflammation, Strabismus, and Appearance) grading system represents yet another classification system to assess clinical activity and severity; the associated VISA Inflammatory Index is scored out of 10 such that a score < 4/10 represents moderate disease and is managed conservatively, and severe disease with a score > 5/10 is managed more aggressively [43,67,79]. Patients with evidence of disease progression and a score of 5 out of 10 are as well managed more aggressively. The EUGOGO severity grading system is more commonly used in Europe, whereas the VISA grading system is more commonly used in North America [59,79].

### 4.3. Imaging Modalities

#### 4.3.1. Computed Tomography

**Figure 4:**
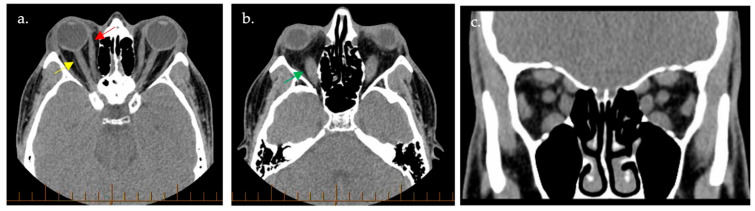
Computed tomography images demonstrated extraocular muscle enlargement in thyroid eye disease. Computed tomography (CT)-based images in axial (**a**,**b**) and coronal (**c**) planes showcase superior (yellow arrow), medial (red arrow), and inferior (green arrow) rectus muscle enlargement bilaterally.

Radiographic imaging is instrumental in guiding the management of TED (Figure 4). Computed tomography (CT) of the orbits classically demonstrates a tendon-sparing enlargement of the EOM bellies, colloquially dubbed the ‘coca-cola sign’, with an increase in the orbital fat volume [53,59,65]. CT imaging may be especially helpful in clarifying the diagnosis of TED by revealing bilateral radiographic disease in cases where the presence of only unilateral disease is suspected clinically [59]. The order of muscle involvement is inferior rectus, medial rectus, and finally, the superior rectus; involvement of the lateral rectus or oblique muscles is not typically seen. Although this is the most frequent finding, it is worth noting that any single or combination of muscle involvement can occur. Other signs include apical crowding and increased angle of the orbital apex (i.e., a deviation from the normal positioning or the orbital apex, which is the point where the optic nerve enters the orbit), increased angle of the medial orbital wall (i.e., the medial orbital wall angles are usually parallel to each other), enlarged superior ophthalmic vein (SOV), orbital fat prolapse, perineural fat effacement, enlarged lacrimal gland, and a taut optic nerve in cases with significant proptosis. Orbitopathy may be radiographically classified as primarily lipogenic (type I), primarily myogenic (type II), or mixed (type III) [59]. CT imaging exophthalmometry should also be measured, as it is more reliable than Hertel exophthalmometry; in cases of lateral orbital decompression where the interzygomatic method of exophthalmometry cannot be utilized, the measurement from the posterior clinoid process to the anterior corneal surface may suffice instead [59,80]. 

#### 4.3.2. Magnetic Resonance Imaging

Magnetic resonance imaging (MRI) can provide additional soft tissue resolution, further aiding in the assessment of disease activity [53]. There is a positive correlation between the T1 signal intensity ratio, T1 post-gadolinium signal intensity ratio, and apparent diffusion coefficient with the CAS. T1-weighted images of inactive TED show isointense EOM and facial muscles, with the enhancement of EOMs post-gadolinium. T2-weighted images of inactive TED show hypointense EOMs, whereas active TED has hyperintense EOMs. Barrett’s index, used to assess for the presence of DON, may be derived from CT imaging or MRI—the vertical index is the sum of the vertical muscle diameters divided by the height of the orbit through the optic nerve; the horizontal index is the sum of the horizontal muscle diameters divided by the width of the orbit through the optic nerve. A Barrett’s index greater than 60% is suggestive of DON [59,81]. 

#### 4.3.3. Orbital Color Doppler Imaging

Orbital color doppler imaging (CDI) is emerging as an important radiographic mean to assess the hemodynamic changes that occur with TED [59]. Orbital and ocular perfusion may be sequentially monitored for prognostication and to assess treatment response [69,82]. Blood flow velocities are measured in the SOV, ophthalmic artery (OA), central retinal artery (CRA), and posterior ciliary artery (PCA). The active phase of TED demonstrates high peak systolic and end-diastolic velocities in the OA and CRA. Reduction or reversal in SOV maximum velocity are noted due to elevation in cone pressure following soft tissue expansion in the orbit; these findings, in addition to the CRA-resistivity index, are improved after orbital decompression [69]. Furthermore, changes in choroidal thickness and the retinochoroidal microvasculature have been noted on OCT with and without angiography [69]. 

### 4.4. Serologies and Molecular Biomarkers

TED is associated with hyperthyroidism in 90% of cases, hypothyroidism in 5%, and occurs in euthyroid individuals in the remaining 5% [54]. TED is most commonly linked to GD, which is caused by auto-antibody stimulation of the TSHR with the TSHR-Ab [53]. TSHR-Ab levels correlate with the CAS, representing the only known biomarker for TED and specifically for GO [1,83]. TSHR expression was shown to be elevated in the orbital fat of patients with TED; the activity of TSHR-Ab through TSHR binding stimulates the release of pro-inflammatory cytokines in the orbital tissues, leading to the accumulation of mucopolysaccharides and glycosaminoglycans with resultant soft tissue swelling [84]. TSHR-stimulating immunoglobulins (TSI), a subtype of TSHR-Ab, have also been found to correlate with disease activity and specifically with DON [85,86]. 

Competitive-binding assays and cell-based bioassays may be used to assess different types of TSHR-Ab throughout the disease course (Table 3). Competitive-binding assays provide information on the presence and concentration of TSHR-Ab through the measurement of TSHR-binding inhibitory immunoglobulins (TBII) and are available globally, whereas cell-based bioassays provide functional information through the measurement of TSI, including whether the antibody is stimulatory or inhibitory, and are generally available in large commercial laboratories or academic centers [83,84]. Both TBII and TSI represent subtypes of the TSHR-Ab. The latest, third-generation competitive-binding assays measure the inhibitory ability of TBII on M22, a labeled F(ab)2 human TSHR-stimulating monoclonal antibody, on the TSHR. TSHR-Ab is detected in 95–98% of patients with GD using third-generation competitive binding assays. Cell-based bioassays utilize stably transfected cell lines or chimeric cell lines expressing TSHR to measure the stimulatory activity in patients with GD, with 99–100% sensitivity and 100% specificity values for antibody function [84]. In contrast, anti-thyroid peroxidase (TPO) and anti-thyroglobulin (anti-Tg) antibodies have not shown a correlation with TED [53]. 

**Table 3:** Sensitivity and specificity of current competitive-binding assays and cell-based bioassays for the diagnosis of thyroid eye disease.

	Sensitivity	Specificity	Reference
TSHR-binding inhibitory immunoglobulin assay [87]			
IMMULITE TSI	98.6%	98.5%	[87]
EliA^TM^	96.6%	99.4%	[88]
Elecsys^®^	100.0%	95.3%	[88]
Cell-based bioassays with TSI measurement			
Thyretain^TM^	97.0%	95.9%	[89]

#### Liquid Biopsy: A Novel Approach

Liquid biopsy is a novel technology that has gained great interest in the past few years for disease diagnosis and monitoring given its non-invasive nature, low cost, highly sensitive nature, and the possibility to provide real-time monitoring of diseases [90]. Although the application of liquid biopsy has been thoroughly discussed in the diagnosis and monitoring of cancers [90,91,92], in this section, we discuss the possibility of translating liquid biopsy technology to TED diagnosis and disease monitoring, a tool not yet applicable given the lack of well-defined, sensitive, and proven biomarkers.

Traditionally, liquid biopsy consists of isolating and analyzing tumor-derived moieties, which encompasses circulating tumor cells (CTCs), circulating tumor DNA (ctDNA), tumor-derived extracellular vesicles (EVs), and circulating cell-free RNA (cfRNA) [90]. The role of miRNAs, which are a subtype of cfRNA, has been discussed in the pathogenesis of TED. It was demonstrated that the serological expression of numerous miRNAs, such as miR-130a [93,94], miR-484, and miR-192-5p [95], is altered in TED. The expression of miR-130a was shown to be elevated in patients with TED, and it modulated adipogenesis through the inhibition of AMPK activity [93,94]. Furthermore, miR-103a-3p was shown to be elevated in TGFβ-activated orbital fibroblasts, where it was shown to promote cell proliferation and differentiation through the ERK-Jun N-terminal kinase (JNK) and TGFβ/Smad signaling pathways [96].

Numerous challenges exist for the implementation of liquid biopsies in the diagnosis, management, and treatment response monitoring of TED. A major drawback is the lack of evidence-based biomarkers. Although several cytokines, miRNAs, and protein expression modifications have been reported in TED, the sensitivity in detecting their serological expression through liquid biopsy has yet to be fully established. Furthermore, technical limitations, such as the lack of resources and well-trained technicians, limit the translation of liquid biopsy into clinical practice for TED management. However, with the growing body of literature on this topic, we believe liquid biopsies could offer a rapid method of screening, diagnosis, and disease monitoring for treatment for TED in the near future.

## 5. Current Therapeutic Approaches

The management of TED currently involves a dynamic and multidisciplinary approach. In mild cases, pharmacological treatment may not be required. However, in moderate or severe disease, medical intervention, predominantly pharmacological but also surgical, is required in order to prevent sight-threatening complications. This section aims to discuss the current pharmacological landscape in TED to highlight the uses, advantages, and limitations of current drugs used in TED treatment (Table 4). However, before delving into the drugs, it is important to note that there is no current universal gold-standard treatment for TED nor a consensus on an optimal treatment plan. Thus, current management is guided by individual expert opinion. Studies aimed at establishing a gold-standard treatment approach would be highly beneficial to the field. In 2021, the EUGOGO published a clinical guideline for the management of TED to help address this issue; however, it has not been widely adopted in clinical practice globally [97].

The current first-line treatment is glucocorticoids (GCs). However, biological agents, notably teprotumumab, which was approved in 2020, are increasingly being used as first-line treatment options. One meta-analysis compared these two options, concluding that teprotumumab is more likely to reduce proptosis and diplopia than GCs [113]. However, the lack of direct comparison limits conclusive evidence of clinical superiority, and thus GCs are still widely the preferred first-line treatment in many countries. Furthermore, the greater cost of teprotumumab compared to GCs makes its selection as a first-line treatment less likely in certain countries where the medical financial budget is limited.

Among GCs, there are two clinically relevant therapies: intravenous methylprednisolone (IVMP) and oral prednisone. Both GCs share common mechanisms of action, which include increasing anti-inflammatory processes and suppressing pro-inflammatory aspects of TED [2,114]. Although IVMP is more laborious to administer, it is preferred to oral prednisone, as it has been demonstrated to be more efficacious and with fewer adverse events than oral prednisone in three randomized controlled trials (RCTs) [2,98,101,102,115]. Thus, IVMP is commonly thought of as the gold standard first-line treatment for moderate-to-severe TED.

Teprotumumab has emerged as a promising treatment for moderate-to-severe TED. Teprotumumab is a humanized monoclonal antibody that partially antagonizes IGF-1R by binding its alpha subunit and is demonstrated to be effective in reducing TED clinical activity, as well as reducing the severity of diplopia and proptosis. Teprotumumab was shown to reduce proptosis by 2.31 mm on average (95% confidence interval range 1.17 to 3.45 mm), as well as reduce the CAS to 0 or 1, outlining its great clinical impact on TED [116]. However, its use was shown to induce ototoxicity [117]. Teprotumumab is the first of several promising biologics to be approved for TED and provides an alternative treatment option for patients with moderate-to-severe TED who cannot tolerate or fail to respond to glucocorticoids. Some clinicians now support the use of teprotumumab as first-line treatment, especially in chronic TED, but its clinical use is limited by its cost—which is estimated at USD 360,000 for a 75 kg patient, 5000 times more than intravenous glucocorticoids [118,119]—and a lack of rigorous scientific evidence directly comparing it to IVMP [120].

In refractory cases of TED, there are several second-line treatment options, such as biologics, orbital radiation, and immunosuppressants. Yet, there is a lack of direct evidence supporting one second-line treatment over the other. As a result, the clinical use of these drugs is individualized for the patients through shared decision-making. There are several options a physician can choose from, including immunomodulators, notably tocilizumab targeting IL-6 and rituximab targeting CD-20, both of which will be discussed in more detail. Additionally, there are a multitude of immunosuppressive agents, such as mycophenolate, azathioprine, cyclosporine, methotrexate, and sirolimus, which are used at the discretion of the physician and patient. Altogether, these immunosuppressive agents serve as viable alternatives to IVMP or teprotumumab, but with limited evidence, it is frequently unclear when one specific agent is superior to another.

TED is treated based on severity. Pharmaceutical intervention is considered when TED progresses to moderate, severe, or sight-threatening stages. The absence of medical intervention in mild cases is due to the high frequency of spontaneous resolution. For these patients, it is sufficient to treat local symptoms and monitor the progression carefully [97,121]. However, it is important to note that selenium has been demonstrated to be beneficial in a subset of patients with mild TED [97,122]. Selenium has an important anti-oxidative effect in the thymus, where it exists in high concentrations. RCT evidence supports selenium supplementation for the improvement in quality of life and ophthalmic outcomes in mild cases of TED [2,97,122]. Even though selenium was only studied in mild cases, EUGOGO recommended a 6-month selenium regimen for those with active disease or mild TED due to its positive impact on ophthalmic outcomes [97].

## 6. Novel Therapeutic Approaches

The cutting-edge advances in TED pathogenesis, as well as in the field of pharmaceutics, have led to the establishment of numerous clinical trials over the past years, with a goal of developing targeted immunotherapy for the treatment of TED (Table 5). In the ensuing section, we will cover the novel therapeutic approaches with strong evidence, the upcoming possibilities, and possible strategies that require further investigation.

### 6.1. Teprotumumab and IGF-1R Inhibitors

**Figure 5:**
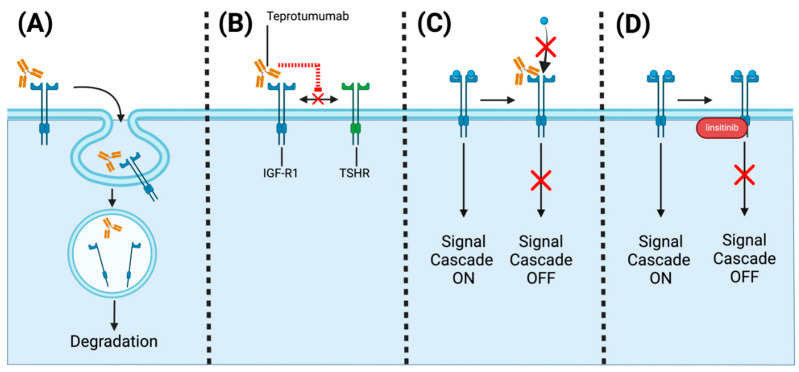
Targeting IGF-R1 for the treatment of thyroid eye disease. (**A**) Tetrotumumab as well as other antibodies against IGF-1R can trigger the internalization and degradation of IGF-1R. (**B**) Antibodies against IGF-1R can disrupt the interaction between TSHR and IGF-1R. (**C**) Antibodies targeting IGF-1R can disrupt ligand binding (red ✕) and downstream signal activation (red ✕). (**D**) Small molecules disrupt the downstream signaling cascade by targeting tyrosine kinase domains (red ✕). The figure was created with BioRender.com.

As discussed in the previous section, teprotumumab is a monoclonal antibody that was initially developed as an oncology drug [2]. However, it was repurposed as a treatment for TED. It functions by partially antagonizing IGF-1R, which co-localizes with TSHR on orbital fibroblasts. By binding IGF-1R’s alpha subunit, teprotumumab inhibits IGF-1R through three mechanisms: (i) internalization and degradation of IGF-1R, (ii) IGF-1R activation blockade, and (iii) IGR-1R and TSHR complex formation inhibition (Figure 5) [123,124,125]. As a result, teprotumumab prevents IGF-1R-mediated activation of IL-6 and IL-8 synthesis by TSH, ultimately having an anti-inflammatory effect [123,124,125]. Teprotumumab is currently administered intravenously, with current regimens requiring patients to receive eight infusions, which are 60 to 90 min in length every 3 weeks, a time-consuming and expensive process [120].

In preliminary phase 2 and 3 OPTIAC trials, teprotumumab was compared to placebo in active moderate and severe TED. The primary endpoint was a decrease in CAS of greater than 2, which was achieved in 69% of patients receiving teprotumumab compared to 20% in the placebo arm at 24 weeks. Along with a strong improvement in quality of life, diplopia, and proptosis (decrease of 2 mm or greater) in patients with GO, these data supported its FDA approval in 2020 for use in TED, independently of its severity [126]. 

Currently, a multi-center phase IV post-marketing study is underway to address the safety of the current regimen and possible alternatives. The study is composed of 300 patients and will test three different cohorts of dosing regimens of teprotumumab (4, 8, or 16 infusions). The study is examining the frequency of treatment-related adverse events and treatment response, which will help clarify the need and clinical use of teprotumumab (NCT05002998).

Teprotumumab, although a promising therapy for TED, is difficult to administer due to its requirement for IV delivery [2,127]. As a result, new IGF-1R antagonists have been sought. VRDN-001 is a full IGF-1R antagonist that was studied in a phase I/II clinical trial at doses of 3 mg/kg, 10 mg/kg, and 20 mg/kg. Key outcomes included an 83% overall responder rate and significant reductions in both proptosis (by 2.4 mm) and CAS (by 4.3 points) [128]. A phase III study is currently being conducted to assess the clinical outcomes of VRDN-001 compared to placebo in patients with moderate to severe TED (NCT05176639). Two other actively recruiting phase III trials are assessing VRDN-001 in specific populations, such as chronic TED patients, and assessing adverse effects over a longer time frame (NCT06021054, NCT06384547). Two new IGF-R1 monoclonal antibodies, VRDN-002 and VRDN-003, are also being studied. They are distinct as they have modified Fc fragments of VRDN-001 to increase the half-life of the drug [129,130,131,132]. In preclinical studies, both VRDN-002 and VRDN-003 were administered to non-human primates intravenously and subcutaneously, with the latter presenting a clinical advantage over teprotumumab. In healthy volunteers, IV administration of VRDN-002 was well tolerated with an increased half-life [130]. The results of these preclinical studies are promising and may fuel future VRDN-002 and VRDN-003 clinical investigations [126]. A notable advantage of these drugs is subcutaneous administration, which is less laborious than IV administration. Lonigutamab is another subcutaneous IGF-1R inhibitor currently in phase I/II clinical study evaluating its safety and efficacy in patients with TED (NCT05683496).

Linsitinib is a small-molecule dual inhibitor of both IGF-1R and insulin receptors. Linsitinib inhibits the tyrosine kinase domain of IGF-1R, preventing autophosphorylation and downstream signaling [133,134]. In preclinical studies in murine models of GO, researchers successfully prevented the development and progression of TED, serving as foundational evidence for clinical studies of linsitinib [133]. A key advantage of linsitinib is its oral route of administration. Currently, a phase IIb RCT (LIDS) is underway to assess the safety and efficacy of linsitinib compared to placebo in patients with active moderate-to-severe TED (NCT05276063). 

### 6.2. Tocilizumab and IL-6 Receptor Antagonists

**Figure 6:**
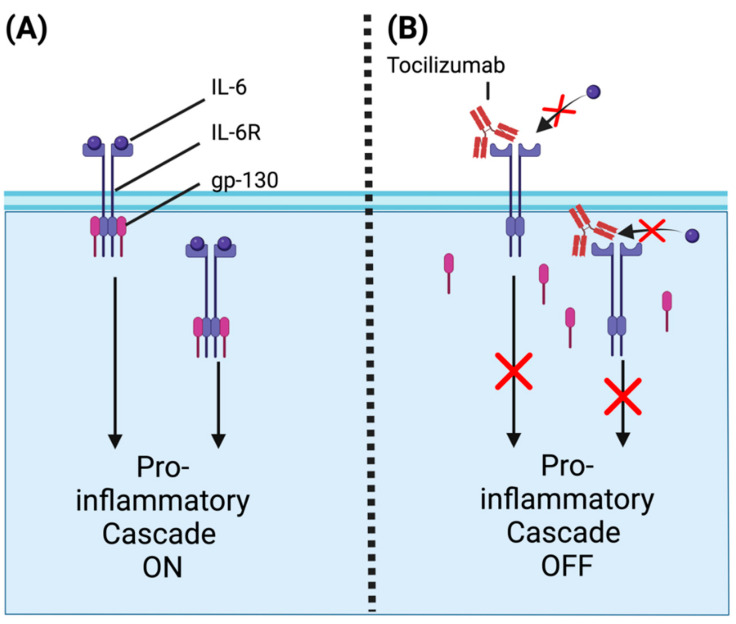
Mechanism of action of antibodies targeting IL-6R. (**A**) Uninhibited IL-6R is activated by its ligand, IL-6, leading to the dimerization of gp-130 and activation of a signaling cascade that activates the pro-inflammatory response. (**B**) Tocilizumab disrupts the interaction between IL-6R and its ligand (red ✕), preventing gp-130 dimerization and inhibiting the signaling cascade that triggers pro-inflammatory responses (red ✕). The figure was created with BioRender.com.

Tocilizumab is a monoclonal antibody widely used in the treatment of autoimmune conditions such as rheumatoid arthritis. It targets interleukin-6 receptors (IL-6R), which recruit gp-130 that then dimerizes to activate a pro-inflammatory cascade involved in B and T cell activation [2]. IL-6R exists both in the soluble and membrane form in a multitude of immune cells, both of which are inhibited by tocilizumab, resulting in the prevention of downstream homodimer formation with gp-130 (Figure 6). The end result is the inhibition of IL-6R-mediated pro-inflammatory pathways such as antibody and acute phase protein synthesis [135].

Prior to its study in TED, tocilizumab was mainly used to treat rheumatoid arthritis. The foundation for its use in TED came from a small RCT (n = 32) with GO patients who had failed IV corticosteroid therapy. In patients receiving IV tocilizumab, there were greater reductions in CAS (86% achieving CAS lower than 3 vs. 39% in the placebo) at 0, 4, 8, and 12 weeks [136]. Other small observational studies, as well as a systematic review, have also supported tocilizumab as a second-line therapy [137,138,139]. However, a larger, long-term RCT would be needed to favor widespread clinical implementation of tocilizumab [137,138,139]. Currently, a phase 2 trial of unknown status is comparing tocilizumab to IVMP in patients with moderate-to-severe TED (NCT04876534), and additional studies would greatly clarify the optimal use of tocilizumab for TED. 

### 6.3. Rituximab and Targeting B Cells

**Figure 7:**
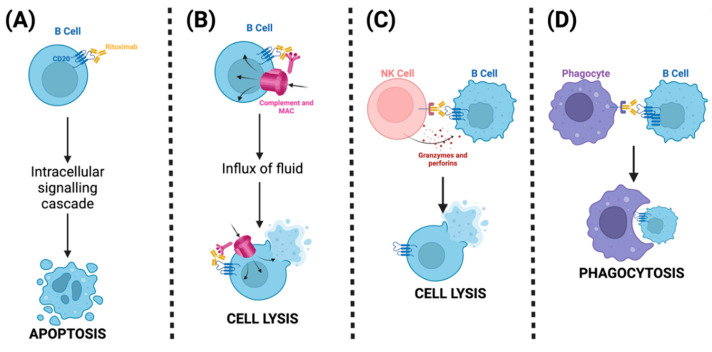
Mechanism of action of Rituximab, an antibody specific to CD20 on the surface of B cells. (**A**) Rituximab binds its ligand CD20, which can trigger an intracellular signaling cascade, leading to apoptosis of B cells. (**B**) Complement activation by rituximab leads to deposition of MACs that cause an influx of fluid and cell lysis of B cells. (**C**) Fc fragments of rituximab trigger Fc receptors on NK cells, activating them and leading to the release of granzymes and perforins and cell lysis of B cells. (**D**) Phagocytes are activated through their Fc receptor, recognizing the Fc fragment of rituximab, leading to the degradation of the B cell through phagocytosis. The figure was created with BioRender.com.

Initially developed as the first molecule to treat non-Hodgkin’s lymphoma, rituximab (RTX) is now increasingly used to treat various autoimmune diseases. RTX is a monoclonal antibody directed against CD20 surface antigen, which is involved in B-cell differentiation and activation [140,141]. RTX inhibits B cells through several pathways. Firstly, RTX activates intracellular signaling pathways, resulting in cell apoptosis (Figure 7) [140,141]. Its Fc domain activates the classical pathway of the complement system by activating C1, leading to the formation of membrane attack complexes (MAC), causing cell lysis [140,141]. Another pathway mediated by RTX’s Fc domain is the activation of NK cells through their fragment crystallizable receptor (FCR), which results in the release of granzymes and perforins, causing cytotoxic death of B cells [140,141]. Finally, through FCRs of phagocytes, such as macrophages, RTX induces phagocytosis of B cells, resulting in their intracellular degradation [140,141].

The use of rituximab to treat TED is based on two RCTs, one conducted in Italy and the other in the United States. However, these two small single-center studies provided conflicting results. In the American study, patients with moderate-to-severe TED were given either RTX or placebo, and no advantage or reduction in CAS was seen in the experimental arm [142]. However, the Italian study compared RTX (1000 mg 2 weeks apart) to IVMP and found that RTX outperformed IVMP, with all patients showing inactivation of GO at 24 weeks in the RTX arm, compared to 69% in the IVMP arm [143]. Despite the conflicting results, RTX is used as a second-line treatment; however, future studies could aim to elucidate which subset of TED patients would benefit most from RTX.

Another strategy to target B-cells has also been investigated. Belimumab, an immunomodulator approved for lupus treatment, is a monoclonal antibody that targets B-lymphocyte stimulator protein (BLyS, aka. BAFF), a cytokine involved in B-cell differentiation [144]. By binding its target BLyS, belimumab inhibits BLyS interaction with BCR as well as other proteins, ultimately inhibiting B-cell differentiation into plasma cells and triggering apoptosis [144]. Belimumab is being studied as a treatment for TED on the basis that BLyS levels are elevated in TED patients. An ongoing RCT comparing belimumab to IVMP is being conducted in Europe in patients with moderate-severe GO (EudraCT Number: 2015-002127-26) [145]. The interim analysis of this study provides evidence that belimumab is as effective as IVMP in treating GO, albeit with a slower effect but notably with improved tolerability [145].

### 6.4. Future Therapeutic Possibilities

#### TSHR Inhibitors

TSHR inhibitors are an emerging, promising therapeutic strategy for TED. This is largely due to past evidence demonstrating that TSHR has a synergistic relationship with IGF-R1 and has been seen to be overexpressed in TED, leading to adipogenesis [2,146]. Even though TSHR’s involvement in TED is not fully understood, drugs targeting it are under study (Table 6).

**Table 6:** Most notable preclinical studies exploring possible novel treatments for thyroid eye disease.

Drug	Mechanism of Action	Results	References
VRDN-002	Antibody targeting IGF-R1	Safely administered to primates intravenously and subcutaneously with increased half-life compared to VRDN-001	[129,130]
VRDN-003	Antibody Targeting IGF-R1	Safely administered to primates intravenously and subcutaneously with increased half-life compared to VRDN-001	[131,132]
S37A	Small molecule that interferes with signal transduction of TSHR	Can inhibit TSHR signaling in the presence of its ligand TSH	[147]
ANTAG3 and Lisitinib	Antibody targeting both TSHR and IGF-R1	A synergistic effect of the drug combination was seen in GO fibroblasts	[148]

In preclinical studies, one such drug, K1-70, a recombinant human antibody, has been shown to interfere with TSHR signaling by interfering with ligand binding [149]. These findings led to a phase 1 clinical trial that demonstrated tolerable IV and intramuscular (IM) administration in patients with Grave’s disease [150]. It is not yet used for TED treatment, as no further clinical trials have yet been conducted. Other TSHR antagonists are being investigated as potential therapies. S37A is a small molecule that interferes with signal transduction by inhibiting TSHR signaling in the presence of its ligand in cell lines and has shown promising results in studies [2,147]. These studies have not yet been translated into practice, but nevertheless, they show that TSHR is a potential target for TED treatments. As TSHR and IGF-R1 cross-communicate, there is an idea to inhibit both targets concurrently. This has been investigated in cultures of GO fibroblasts [148,151]. These studies revealed a synergistic effect, one that could be beneficial to reduce required doses or compensate for the inefficiency of targeting solely one receptor [148]. Further studies are required to conclude if there is any clinical significance of this combination strategy.

### 6.5. Future Therapeutic Strategies

#### 6.5.1. Targeting Cytokines

The promising results of recently FDA-approved immunomodulating agents have further driven the exploration of novel immune markers, cytokines, and cells as targets. Most of the drug candidates have been validated in preclinical models and are currently being investigated in clinical trials. The first notable target being studied is IL-17. IL-17 has been associated with TED pathophysiology, and thus a group is examining vunakizumab (SHR-1314), a subcutaneously administered monoclonal antibody targeting IL-17a, preventing its interaction with its receptor. A phase I study in psoriasis patients demonstrated that vunakizumab is tolerable in human subjects, and this study is being translated into a phase II clinical trial for patients with moderate-to-severe TED (NCT05394857). Another group conducted a phase III clinical trial for secukimumab, another monoclonal antibody targeting IL-17a, in patients with TED (NCT04737330). However, this study was terminated early due to the low probability of meeting the primary endpoint. Therefore, targeting IL-17a as a therapy for TED still remains unconfirmed [152].

Another cytokine being targeted in phase I and II clinical trials in patients with TED and pulmonary fibrosis is IL-11 (NCT05331300, NCT0622545). Although the role of IL-11 in TED is not well understood, it is postulated that IL-11 may play a role in the altered phenotype of orbital fibroblasts [2,153]. Thus, researchers are currently striving to inhibit the IL-11 receptor (IL-11R) with LASN01, a fully human antibody. The goal of this strategy is to prevent signaling and pro-inflammatory activity mediated by IL-11R, which would act as a potential therapy for TED.

#### 6.5.2. Neonatal Fragment Crystallizable Receptor

The neonatal fragment crystallizable receptor (FcRn) plays a crucial role in transporting immunoglobulin G (IgG) across barriers and protecting it from degradation. FcRn inhibitors have recently emerged as a potential treatment for various autoimmune diseases, including Myasthenia Gravis [154]. Batoclimab is a monoclonal antibody that targets FcRn, preventing it from recycling IgG [126]. In TED, this may enhance the breakdown of pathogenic autoantibodies targeting TSHR and IGF-R1 [126]. In a phase IIa trial (ASCEND-GO 1), seven patients with active moderate-to-severe TED received batoclimab as weekly subcutaneous injections. The results showed a 64.8% decrease in serum IgG levels and a 56.7% reduction in anti-TSHR antibodies [155]. However, the subsequent phase IIb trial (ASCEND-GO 2) was terminated due to increased serum cholesterol levels in participants [2,126]. It is unclear if batoclimab will be used in TED clinically. To address this, a phase III, multi-center, randomized, placebo-controlled study is currently ongoing to evaluate the efficacy of batoclimab for the treatment of TED (NCT05517421, NCT0552457).

#### 6.5.3. Sirolimus

Sirolimus is an immunosuppressive agent that has recently been studied in clinical trials to treat TED. It acts by suppressing the sensitivity of lymphocytes to cytokines, predominantly to IL-2, thus reducing lymphocyte activation [2]. In a preliminary observational study comparing sirolimus to IVMP, the sirolimus cohort outperformed IVMP at 24 weeks (86.6% to 26.6%) [109]. This study subsequently fueled a phase II trial, comparing first-line use of sirolimus to IVMP in patients with GO, striving to provide additional evidence to clarify the clinical significance of sirolimus (NCT04598815). 

#### 6.5.4. Statins

Statins are used to reduce cholesterol levels by inhibiting 3-hydroxy-3-methylglutaryl-coenzyme A (HMG-CoA). In recent studies, statins have been shown to be effective in treating TED. This is due to their anti-inflammatory and anti-immunomodulatory properties. Their anti-inflammatory effects include inhibiting the induction of pro-inflammatory cytokines released from T lymphocytes. One recent phase II clinical trial evaluated the effects of adding atorvastatin to intravenous glucocorticoids in patients with GO and hypercholesterolemia. Results showed that adding a statin to IVMP improved outcomes in patients with GO, although a bigger population is needed to confirm the results found in this study [156]. 

## 7. Novel Advances in Artificial Intelligence for the Management of Thyroid Eye Disease

Artificial intelligence (AI) is rapidly becoming immersed in medical practices. Its application within the field of ophthalmology has grown quickly as it presents the possibility to optimize diagnostics, prognostics, and disease management. TED is a highly complex and variable disease, reliant on a thorough diagnosis from imaging, clinical assessment, and thyroid function assessment. AI can be a valuable tool in managing TED, aiding every step of the way, from diagnosis and tracking disease progression to personalizing treatments. 

### 7.1. Diagnostic Applications

Traditionally, TED diagnosis relies on a physician’s expertise, which can lead to inconsistencies in diagnosis and can limit the early detection of disease [157]. To address current limitations, AI modalities are being developed to facilitate the diagnosis and screening of TED through the accurate identification of early-stage clinical markers of TED. For example, current methods of measuring proptosis are limited by their lack of reproducibility and unreliability, largely due to the high degree of subjectivity involved [158]. To objectify this measurement, one study developed an AI model that can quickly and reliably identify clinically relevant proptosis based on orbital CT scans. Their model measured proptosis at a concordance correlation coefficient (CCC) of 0.9895 on axial CT images and a CCC of 0.9902 on sagittal CT images, which were both comparable to the results of clinician-based measurements [158]. With more advancements, an improved model would be a promising alternative to current standards that would optimize the reliability and accuracy of proptosis measurements. Another group applied their machine learning model to facial scans to assess the facial symptoms of TED. They trained their model to recognize specific symptoms, including but not limited to eyelid retraction, ocular dyskinesia, and conjunctival congestion. They concluded that their model could reliably identify patients with facial symptoms of TED with an average area under the curve (AUC) of 0.85 [159]. 

Beyond identifying and quantifying symptoms of TED, AI-based modalities have been explored for the purpose of screening for TED. One study developed an AI-powered tool aimed at diagnosing TED without clinician input based on orbital CT scans [160]. The model was externally verified (AUC of 0.919), and through non-inferiority experiments, the researchers concluded that their model could diagnose TED (85.67%) as accurately as residents (84.33%), whose clinical interpretation is the current benchmark in TED diagnosis [160]. If such a model could be perfected to outperform clinicians, they could be used to screen patients at risk, providing a valid, reliable, and automated diagnostic tool [157,160].

### 7.2. Disease Monitoring

AI could also aid in the management of TED by quantifying the severity of the disease to track progression and assist in the selection of an optimal treatment plan. To explore this application, one study developed an AI-powered program that could quantify inflammation of extraocular muscles based on contrast-enhanced MRI in order to evaluate the activity stage of TED [161]. The study examined three machine learning models, and the light gradient boosting machine (LightGBM) had the best diagnostic classification performance with an AUC of 0.9260 [161]. Another study examined a machine learning model’s ability to assess CAS. They found that their model could predict the CAS based on patients’ facial scans with an accuracy of 84.6% when training the model with an unfiltered data set, and the accuracy increased to 89.0% when trained with filtered data sets with consistent results [162]. Additionally, an AI-based model was trained off orbital CT scans to differentiate between active and inactive TED, achieving an AUC of 0.871 [163]. Altogether, these studies all developed AI-based modalities with the potential to be used clinically to analyze images to quickly provide clinicians with clinically relevant information that could assist in tracking disease progression and activity. 

### 7.3. Decision Making Tool

The information acquired from AI-driven systems can also help clinicians guide their treatment selection. This was demonstrated by a group of researchers whose convolutional neural network (CNN), specifically tailored for image-centric tasks, could analyze orbital CT scans to quantify proptosis and identify patients that required surgical drainage [164]. Although this model’s measurements of proptosis differed significantly from those made by physicians, it still showed promise, as the linear and volumetric measurements of proptosis made by the CNN were more predictive of the requirement of surgery (AUC of 0.78 and 0.79, respectively) than physician measurements (AUC of 0.70) [164]. The volumetric AI-based proptosis measurements from CNN could be a valuable clinical tool to predict the likelihood of surgery. Another group examined the use of AI models in predicting TED patients’ responses to treatment. Their model, XGBoost, analyzed clinical characteristics and laboratory results, predicting the response of TED patients to steroid treatment with an accuracy of 0.861 [52]. When investigating the factors most impactful on XGBoost performance, the researchers determined that specific patterns of both thyroid-stimulating immunoglobulin and low-density lipoprotein cholesterol were the most significant in predicting the XGBoost’s ability to predict response [52]. These results further demonstrate the use of AI-based modalities in identifying specific clinical patterns that can impact a patient’s response to treatment or guide a physician in choosing the optimal treatment.

Altogether, the data that the AI tool can acquire from facial scans, imaging modalities (e.g., CT scans and MRIs), and laboratory diagnostics can open the possibility of quicker and improved clinical decision-making. Recent efforts to integrate AI in the clinical management of TED have demonstrated its potential to improve diagnosis, progression assessment, and clinical decision-making. As these systems continue to advance, their clinical integration will grow, offering clinicians valuable tools to deliver optimal and comprehensive care. However, major challenges constitute drawbacks to the translation of AI-based deep learning models for the management of TED to clinical practice [157]. The complexity of TED and the variability in its clinical manifestations are significant barriers. Greater training data sets are required to achieve excellent performance of deep learning models and enable their generalization to different populations. Furthermore, such models come with a great cost—high costs in their development and future application to clinical medicine. Additional technical limitations are also faced, such as the ethical aspects surrounding data privacy and the necessity for validation steps to filter and manage potential AI-derived medical errors.

## 8. Current Challenges and Future Perspectives

Autoimmune diseases, such as TED, have strikingly varying phenotypes and encompass complex pathogenesis. A current limitation in the management of TED is the lack of reliable markers to diagnose, assess the severity of disease, and establish treatment response [108]. Currently, ocular examination, repetitive laboratory investigations, and imaging modalities form the backbone of TED diagnosis. However, numerous efforts have been deployed in recent years to develop less invasive, cost-friendly, and rapid diagnostic tools such as liquid biopsy and AI-derived deep learning models. However, their translation to clinical practice is yet to be optimized. The limited understanding of disease pathology currently limits the exploitation of biological agents for TED treatment. For example, although TED patients do have IGF-1R autoantibodies, the presence of such markers has not been associated with the severity of the disease nor with susceptibility to specific treatments [108]. Further studies are thus required to close the fundamental science knowledge gap regarding TED pathogenesis. 

Management of patients with TED involves a multidisciplinary approach that may require input from several specialists. Currently, the lack of consensus on the optimal treatment plan results in varying treatment approaches. IVMP and GCs have been used as the gold standard, followed by immunosuppressive agents. However, new biologics have emerged that may offer enhanced therapeutic benefits with fewer adverse events [108]. Teprotumumab is now viewed as a first-line treatment in TED patients in the United States [165], but its use is limited worldwide in the same setting, given its greater cost compared to GCs. We believe that through rigorous studies and the development of alternative options by pharmaceutical companies, these medications will offer specialists more diverse options to combat TED. Overall, biological agents will be most effective in an era of personalized medicine, whereby specific diagnostic markers can guide clinicians to optimal treatment regimens. Thus, linking specific markers to susceptibility to biologics may be required to fully reach the potential of biologics in treating TED.

## Figures and Tables

**Table 1:** Summary of the required ocular eye exam assessments and their possible findings suggestive of thyroid eye disease.

Ocular Examination Component	Findings
Examination of the orbits and adnexa	
Eyelids	Presence of lateral flare, lid lag, lagophthalmos and Bell’s reflex, upper and lower eyelid retraction
Extraocular muscle assessment	Extraocular eye movement limitations Presence of proptosis secondary to extraocular muscle enlargement
Anterior segment examination	
Cornea	Decreased tear breakup time Superior limbic keratoconjunctivitis Exposure keratopathy Corneal ulceration
Posterior segment examination	
Optic nerve assessment	Increased cup-to-disc ratio Optic nerve head pallor Optic disc swelling Decreased color vision Presence or absence of a relative afferent pupillary defect
Neurological investigations	
Visual field testing	Presence of any visual field defect secondary to optic nerve damage

**Table 2:** Summary of classification systems for clinical activity and severity of thyroid eye disease.

Grade	Criteria
*NOSPECS* ^a^ system	
0	No physical signs or symptoms
I	Only signs, no symptoms
II	Soft tissue involvement
II-0	Absent
II-1	Minimal
II-2	Moderate
II-3	Marked
III	Proptosis (>2 mm of normal upper limit)
III-1	3–4 mm
III-2	5–7 mm
III-3	>8 mm
IV	Extraocular muscle involvement
IV-0	Absent
IV-1	Limitation of motion at extremes of gaze
IV-2	Evident restriction of movements
IV-3	Fixed globe(s)
V	Corneal involvement due to lagophthalmos
V-0	Absent
V-1	Stippling of cornea
V-2	Ulceration
V-3	Clouding, necrosis, and perforation
VI	Sight loss due to optic nerve involvement
VI-0	Absent
VI-1	Disc pallor or visual field defect Visual acuity 20/20 to 20/60
VI-2	Disc pallor or visual field defect Visual acuity 20/70 to 20/200
VI-3	Blindness Visual acuity less than 20/200
*Mild disease encompasses grades 1 to 3, whereas severe disease encompasses grades 4 to 6.*	
*RELIEF* ^a^ system	
R	Resistance to retropulsion
E	Edema of conjunctiva and caruncle
L	Lacrimal gland enlargement
I	Edema of eyelids
F	Fullness of eyelids
Clinical activity score	
Initial evaluation scored out of 7	
	Spontaneous orbital pain
	Gaze-evoked orbital pain
	Lid edema
	Lid erythema
	Conjunctival erythema
	Chemosis
	Caruncle or plica inflammation
Successive evaluations scored out of 10	
	Increase of >2 mm proptosis
	>8° decrease in ocular motility of one eye in any direction
	Decrease in visual acuity of 1 Snellen line
*A clinical assessment score (CAS) greater than 3 out of 7 or 4 out of 10 is considered to be an active disease. Each positive element is given one point.*	
European Group of Graves’ Orbitopathy severity grading system	
Soft tissue assessment	
	Eyelid swelling
1	Absent
2	Mild: none of the features defining moderate or severe categories
3	Moderate: definite swelling, no lower eyelid festoons or angulation of the upper eyelid skin fold in downgaze
4	Severe: lower eyelid festoons or upper eyelid fold becomes rounded at 45° in downgaze
	Eyelid erythema
1	Absent
2	Present
	Conjunctival erythema
1	Absent
2	Mild: equivocal or minimal
3	Moderate: <50%
4	Severe: >50%
	Conjunctival edema
1	Absent
2	Present
	Caruncle or plica semilunaris inflammation
1	Absent
2	Present
Document	
	Lid margin assessment at the mid-pupillary line
	Palpebral aperture (mm)
	Upper and lower lid retraction (mm)
	Levator function (mm)
	Lagophthalmos
1	Absent
2	Present
	Bell’s phenomenon
1	Absent
2	Present
Proptosis assessment	
	Hertel exophthalmometry to record intercanthal distance and amount of proptosis
Ocular motility assessment	
	Prism cover test
	Monocular ductions
	Head posture
	Torsion
	Binocular single vision
Corneal integrity assessment	
	Normal
	Punctate keratopathy
	Ulcer
	Perforation
Optic nerve assessment	
	Visual acuity
	Color vision
	Visual field analysis
	Optic disc assessment: normal, atrophied, glaucomatous, or edematous
	Afferent pupil defect
1	Present
2	Absent
*Mild disease encompasses one or more of the following: minor lid retraction (<2 mm), mild soft-tissue involvement, exophthalmos < 3 mm above normal for race and gender, no or intermittent diplopia, and corneal exposure responsive to treatments; moderate-to-severe disease encompasses two or more of the following: lid retraction > 2 mm, moderate or severe soft-tissue involvement, exophthalmos > 3 mm above normal for race and gender, inconsistent or consistent diplopia; sight-threatening disease encompasses patients with dysthyroid optic neuropathy and/or corneal breakdown.*	
*VISA* ^a^ system	
1 point	Vision
	Visual acuity
	Pupillary reflex
	Color vision
	Visual fields
	Optic nerve examination
	Visual evoked potentials
10 points	Inflammation/congestion
	Caruncular edema
0	Absent
1	Present
	Chemosis
0	Absent
1	Conjunctiva lies behind the gray line of the lid
2	Conjunctiva extends anterior to the gray line of the lid
	Conjunctival erythema
0	Absent
1	Present
	Lid redness
0	Absent
1	Present
	Lid edema
0	Absent
1	Present, without redundant tissues
2	Present, with lower lid festoon and bulging palpebral skin
	Retrobulbar ache at rest
0	Absent
1	Present
	Retrobulbar ache with gaze
0	Absent
1	Present
	Diurnal variation
0	Absent
1	Present
6 points	Strabismus/motility restriction
	Diplopia
0	Absent
1	Diplopia with horizontal or vertical gaze
2	Intermittent diplopia in primary gaze
3	Constant diplopia in primary gaze
	Ocular restriction
0	Duction > 45°
1	Duction 30–45°
2	Duction 15–30°
3	Duction < 15°
3 points	Appearance/exposure
	Appearance concerns (i.e., proptosis, lid retraction, and fat pockets)
	Symptoms from ocular exposure (i.e., foreign body sensation, photophobia, dryness, and secondary tearing)
	Measurements including eyelid retraction, scleral show, LPS function, lagophthalmos, and proptosis with Hertel exophthalmometer
	Signs of corneal exposure, including punctate epithelial erosions, ulcerations, corneal thinning, and corneal perforation
The VISA classification system tool is useful for assessing disease progression or response to therapy, as well as disease activity or quiescence.	

^a^ Abbreviations: NOSPECS, No signs or symptoms, Only signs, no symptoms, Soft tissue involvement, Proptosis, Extraocular muscle involvement, Corneal involvement, Sight loss; RELIEF, Resistance to retropulsion, Edema of the conjunctiva and caruncle, Lacrimal gland enlargement, Injection of horizontal rectus muscle insertions, Edema of the eyelids, and Fullness of the eyelids; VISA, Vision, Inflammation, Strabismus, and Appearance.

**Table 4:** A summary of the posology, advantages, and disadvantages of current immunosuppressive and glucocorticoids used to treat thyroid eye disease.

Treatment	Posology	Advantages	Disadvantages	References
First-line treatments				
Intravenous Methylprednisolone (IVMP)	EUGOGO Regimen ▪ 6 weekly infusions of 500 mg ▪ In moderate to severe disease, an additional 6 weekly infusions of 250 mg ▪ Doses can be adjusted between 4.5 and 7.5 g/cycle according to severity	▪ Gold standard: effectively controls disease activity. ▪ Induces rapid anti-inflammatory response ▪ Higher response rates compared to oral prednisone ▪ Well tolerated with fewer reported serious adverse effects compared to oral steroids	▪ IV administration ▪ Safety concerns with cumulative high doses (>8 g per cycle) ▪ Monitoring for adverse events, including arrhythmias, hyperglycemia, infections, etc. ▪ Requires gastric and bone protection	[97,98,99,100,101]
Oral Prednisone	▪ 0.5 to 1 mg/kg/day tapered based on response ▪ Lowest effective dose over prolonged periods	▪ Oral administration ▪ Effective in mild to moderate TED	▪ Slower onset of action ▪ Higher risk of systemic adverse effects than IVMP	[102]
Second-line treatments				
Azathioprine	▪ Initial dose of 1 to 2 mg/kg/day orally, in combination with radiotherapy or corticosteroids	▪ Can reduce risk of relapse following steroid taper ▪ Beneficial outcomes in combination with glucocorticoids	▪ Only effective as a combination therapy ▪ Adverse effects: bone marrow suppression, nausea, vomiting	[103,104,105]
Cyclosporine	▪ 2 to 5 mg/kg/day orally in combination with prednisone	▪ Fast onset of action ▪ Effective in combination therapy with prednisone	▪ Narrow therapeutic index requiring therapeutic drug monitoring ▪ Serious adverse effects: nephrotoxicity, hypertension, neurotoxicity	[106,107,108]
Mycophenolate	▪ Mycophenolate mofetil 1 g daily orally or mycophenolate sodium 0.72 g daily orally for 24 weeks in combination with IVMP	▪ Improved clinical outcomes when used in combination with IVMP ▪ Less severe adverse effects than corticosteroids	▪ Requires monitoring for gastrointestinal disturbances and systemic adverse effects ▪ Lack of long-term data	[2]
Sirolimus	▪ 2 mg orally on Day 1 followed by 0.5 mg/day for 12 weeks	▪ Superior response rate compared to IVMP in moderate-to-severe TED ▪ Anti-fibrotic properties in renal and pulmonary transplant patients	▪ Significant adverse effect profile requiring careful monitoring	[109]
Methotrexate	▪ Typically initiated at 7.5 to 10 mg orally weekly	▪ Effective in reducing inflammation by suppressing lymphocytes ▪ Promising results in combination with glucocorticoids for sight-threatening TED	▪ Lack of robust randomized controlled trials (RCTs) limits evidence of efficacy ▪ Potential adverse effects include fatigue, nausea, and hair loss	[110,111,112]

**Table 5:** An overview of the current and recently completed clinical trials investigating novel treatments for thyroid eye disease.

NCT Number (Start-End Date)	Study Phase	Drug	Mechanism of Action	Study Title
NCT04598815 (2023–2025)	Phase II	Sirolimus	Inhibits T cell proliferation and fibroblast proliferation	Sirolimus for Graves’ Orbitopathy (GO) (SIRGO)
NCT04936854 (2023–2026)	Phase II	Sirolimus	Immunosuppression: inhibits T-cell activation, inhibits the IGF-1 pathway, anti-fibroblast effects	Sirolimus vs. Corticosteroids in Treatment of Thyroid Eye Disease
NCT05126147 (2022–2024)	Phase IV	Hydroxychloroquine	Inhibits proliferation and adipogenesis in orbital fibroblasts	Hydroxychloroquine in Mild Graves’ Orbitopathy
NCT04359979 (2020–2023)	N/A	Tamsulosin	Inhibits catecholamine alpha-1 receptors	Tamsulosin for Thyroid Lid Retraction
NCT05394857 (2022–2023)	Phase II	Vunakizumab	Antibody targeting IL-17a, preventing interaction with receptor	Efficacy and Safety of SHR-1314 by Subcutaneous Injection in Active Moderate to Severe Graves’ Orbitopathy Patients
NCT04737330 (2021–2023)	Phase III	Secukinumab	Antibody targeting IL-17a, preventing interaction with receptor	A Study of the Efficacy and Safety of Secukinumab 300 mg in Patients With Thyroid Eye Disease (TED) (ORBIT)
NCT05331300 (2022–2025)	Phase I/II	LASN01	Antibody against IL-11R	A Study to Evaluate the Safety, Preliminary Efficacy, and Pharmacokinetic Properties of LASN01 in Healthy Subjects and in Patients With Pulmonary Fibrosis or Thyroid Eye Disease
NCT05002998 (2021–2025)	Phase IV	Teprotumumab	Antibody that inhibits IGF-1R	TEPEZZA^®^ (Teprotumumab-trbw) Post-Marketing Requirement Study
NCT05276063 (2022–2026)	Phase IIb	Linsitinib	Small molecule that Inhibits intrinsic tyrosine kinase activity of IGF-1R and IR	A Phase 2b, Study of Linsitinib in Subjects With Active, Moderate to Severe Thyroid Eye Disease (LIDS)
NCT05176639 (2021–2023)	Phase I, II, III	VRDN-001	Antibody that Inhibits IGF-1R	A Safety, Tolerability and Efficacy Study of VRDN 001 in Healthy Volunteers and Persons With Thyroid Eye Disease (THRIVE)
NCT06021054 (2023–2025)	Phase III	VRDN-001	Antibody that Inhibits IGF-1R	A Randomized, Double-masked, Placebo-controlled Safety, Tolerability, and Efficacy Study of VRDN-001, a Humanized Monoclonal Antibody Directed Against the IGF-1 Receptor, in Participants With Chronic Thyroid Eye Disease (TED) (THRIVE-2)
NCT06384547 (2024–2026)	Phase III	VRDN-001	Antibody that Inhibits IGF-1R	A Randomized, Active Controlled, Safety and Tolerability Study of VRDN-001 in Participants With Thyroid Eye Disease (TED) (STRIVE)
NCT05683496 (2023–2024)	Phase I-II	Lonigutamab	Inhibits IGF-1R	A Phase 1/2, Adaptive, Multiple Dose Ranging Study Evaluating the Safety, Tolerability, Pharmacokinetics, and Clinical Efficacy of Lonigutamab in Subjects With Thyroid Eye Disease (TED)
NCT04876534 (2022–2023)	Phase II	Tocilizumab	Antibody that inhibits membrane-bound and soluble IL-6R	Tocilizumab in Active Moderate-severe Graves’ Orbitopathy (TOGO)
NCT05987423	Phase III	Satralizumab	Humanized monoclonal antibody targeting IL-6R	Study To Evaluate The Efficacy, Safety, Pharmacokinetics, And Pharmacodynamics Of Satralizumab In Participants With Moderate-To-Severe Thyroid Eye Disease
NCT02378298 (2011–2024)	Phase IV	Rituximab	Antibody that inhibits CD-20 on B lymphocytes	Rituximab (RTX) Therapy in Patients With Active TAO
EudraCT Number: 2015-002127-26 (2015-ongoing)	Phase II	Belimumab	Antibody targeting BLyS resulting in the inhibition of B cell differentiation and triggering apoptosis	Comparison between treatment with belimumab and methylprednisolone in Graves’hyperthyroidism (GD) and active orbitopathy (GO).
NCT05517421, NCT0552457 (2022–2025)	Phase III	Batoclimab	Inhibits binding of FcRn to anti-TSHR autoantibodies, leading to degradation of anti-TSHR autoantibodies	Study to Assess Batoclimab in Participants With Active Thyroid Eye Disease
